# Neural Plasticity in Moderate to Severe Chronic Stroke Following a Device-Assisted Task-Specific Arm/Hand Intervention

**DOI:** 10.3389/fneur.2017.00284

**Published:** 2017-06-14

**Authors:** Kevin B. Wilkins, Meriel Owen, Carson Ingo, Carolina Carmona, Julius P. A. Dewald, Jun Yao

**Affiliations:** ^1^Department of Physical Therapy and Human Movement Sciences, Northwestern University, Chicago, IL, United States; ^2^Northwestern University Interdepartmental Neuroscience, Northwestern University, Chicago, IL, United States; ^3^Department of Biomedical Engineering, Northwestern University, Chicago, IL, United States; ^4^Department of Physical Medicine and Rehabilitation, Northwestern University, Chicago, IL, United States

**Keywords:** stroke, hand rehabilitation, EEG, cortical reorganization, voxel-based morphometry, functional electrical stimulation, gray matter, neuroplasticity

## Abstract

Currently, hand rehabilitation following stroke tends to focus on mildly impaired individuals, partially due to the inability for severely impaired subjects to sufficiently use the paretic hand. Device-assisted interventions offer a means to include this more severe population and show promising behavioral results. However, the ability for this population to demonstrate neural plasticity, a crucial factor in functional recovery following effective post-stroke interventions, remains unclear. This study aimed to investigate neural changes related to hand function induced by a device-assisted task-specific intervention in individuals with moderate to severe chronic stroke (upper extremity Fugl-Meyer < 30). We examined functional cortical reorganization related to paretic hand opening and gray matter (GM) structural changes using a multimodal imaging approach. Individuals demonstrated a shift in cortical activity related to hand opening from the contralesional to the ipsilesional hemisphere following the intervention. This was driven by decreased activity in contralesional primary sensorimotor cortex and increased activity in ipsilesional secondary motor cortex. Additionally, subjects displayed increased GM density in ipsilesional primary sensorimotor cortex and decreased GM density in contralesional primary sensorimotor cortex. These findings suggest that despite moderate to severe chronic impairments, post-stroke participants maintain ability to show cortical reorganization and GM structural changes following a device-assisted task-specific arm/hand intervention. These changes are similar as those reported in post-stroke individuals with mild impairment, suggesting that residual neural plasticity in more severely impaired individuals may have the potential to support improved hand function.

## Introduction

Nearly 800,000 people experience a new or recurrent stroke each year in the US (1). Popular therapies, such as constraint-induced movement therapy (CIMT), utilize intense task-specific practice of the affected limb to improve arm/hand function in acute and chronic stroke with mild impairments (2, 3). Neuroimaging results partially attribute the effectiveness of these arm/hand interventions to cortical reorganization in the ipsilesional hemisphere following training in acute and mild chronic stroke (4). Unfortunately, CIMT requires certain remaining functionality in the paretic hand to execute the tasks, and only about 10% of screened patients are eligible (5), thus disqualifying a large population of individuals with moderate to severe impairments. Recently, studies using device-assisted task-specific interventions specifically targeted toward moderate to severe chronic stroke reported positive clinical results (6–8). However, these studies primarily focus on clinical measures, but it is widely accepted that neural plasticity is a key factor for determining outcome (9–11). Consequently, it remains unclear whether moderate to severe chronic stroke [upper extremity Fugl-Meyer Assessment (UEFMA) < 30] maintains the ability to demonstrate neural changes following an arm/hand intervention.

Neural changes induced by task-specific training have been investigated widely using animal models (12). For instance, monkeys or rodents trained on a skilled reach-to-grasp task express enlarged representation of the digits of the hand or forelimb in primary motor cortex (M1) following training as measured by intracortical microstimulation (13, 14). Additionally, rapid local structural changes in the form of dendritic growth, axonal sprouting, myelination, and synaptogenesis occur (15–18). Importantly, both cortical and structural reorganization corresponds to motor recovery following rehabilitative training in these animals (19, 20).

The functional neural mechanisms underlying effective task-specific arm/hand interventions in acute and chronic stroke subjects with mild impairments support those seen in the animal literature described above. Several variations of task-specific combined arm/hand interventions, including CIMT, bilateral task-specific training, and hand-specific robot-assisted practice, have shown cortical reorganization such as increased sensorimotor activity and enlarged motor maps in the ipsilesional hemisphere related to the paretic arm/hand (21–24). These results suggest increased recruitment of residual resources from the ipsilesional hemisphere and/or decreased recruitment of contralesional resources following training. Although the evidence for a pattern of intervention-driven structural changes remains unclear in humans, several groups have shown increases in gray matter (GM) density in sensorimotor cortices (25), along with increases in fractional anisotropy in ipsilesional corticospinal tract (CST) (26) following task-specific training in acute and chronic stroke individuals with mild impairments.

The extensive nature of neural damage in moderate to severe chronic stroke may result in compensatory mechanisms, such as contralesional or secondary motor area recruitment (27). These individuals show increased contralesional activity when moving their paretic arm, which correlates with impairment (28, 29) and may be related to the extent of damage to the ipsilesional CST (30). This suggests that more impaired individuals may increasingly rely on contralesional corticobulbar tracts such as the corticoreticulospinal tract to activate the paretic limb (29). These tracts lack comparable resolution and innervation to the distal parts of the limb, thus sacrificing functionality at the paretic arm/hand (31). Since this population is largely ignored in current arm/hand interventions, it is unknown whether an arm/hand intervention for these more severely impaired post-stroke individuals will increase recruitment of residual ipsilesional corticospinal resources. These ipsilesional CSTs maintain the primary control of hand and finger extensor muscles (32) and are thus crucial for improved hand function. Task-specific training assisted by a device may reengage and strengthen residual ipsilesional corticospinal resources by training distal hand opening together with overall arm use.

The current study seeks to determine whether individuals with moderate to severe chronic stroke maintain the ability to show cortical reorganization and/or structural changes alongside behavioral improvement following a task-specific intervention. We hypothesize that following a device-assisted task-specific intervention, moderate to severe chronic stroke individuals will show similar functional and structural changes as observed in mildly impaired individuals, demonstrated by (i) a shift in cortical activity related to paretic hand opening from the contralesional hemisphere toward the ipsilesional hemisphere and (ii) an increase in GM density in sensorimotor cortices in the ipsilesional hemisphere.

## Materials and Methods

### Subjects

**Figure 1:**
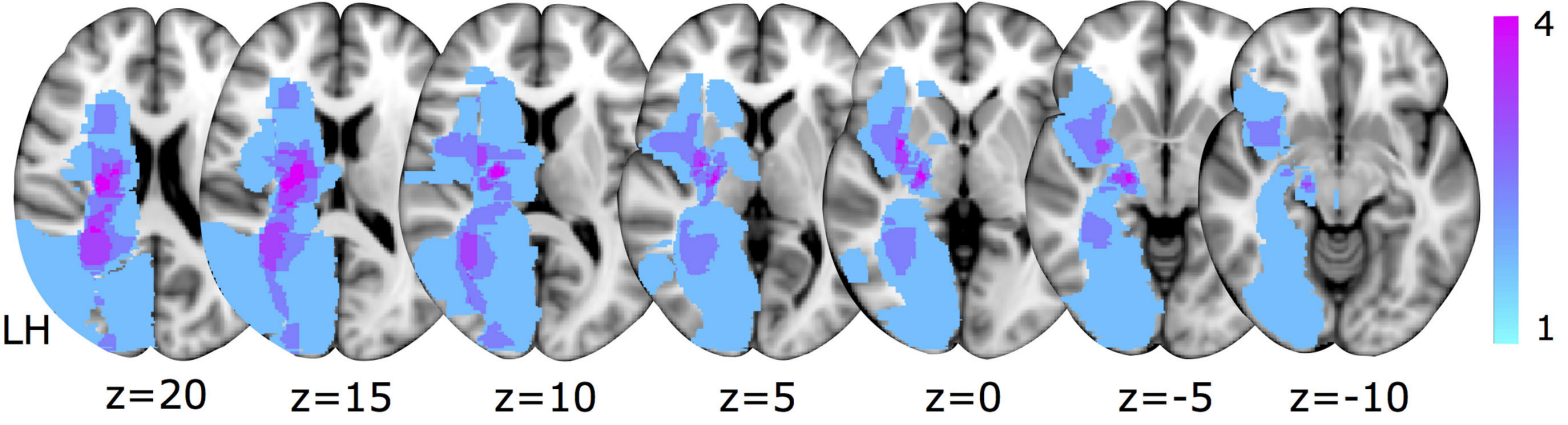
Lesion locations for the eight subjects overlaid on axial Montreal Neurological Institute T1 slices. The color bar indicates the number of subjects with lesioned tissue in a particular voxel. LH indicates the lesioned hemisphere.

Eight individuals with chronic hemiparetic stroke (age: 63.5 ± 4) and moderate to severe impairment (UEFMA: 11–24) participated in this study. Clinical information for each subject is provided in Table 1 and lesion locations in Figure 1. All individuals were screened for inclusion by a licensed physical therapist. Inclusion criteria include a UEFMA between 10 and 30 out of 66, no cognitive or perceptual impairment, no botulinum toxin within the last 6 months, MRI compatibility, no lesion including sensorimotor cortices, the ability to elicit enough EMG activity at wrist/finger extensors, and the ability for the FES to generate a hand opening of at least 4 cm between the thumb and the index finger. This study was approved by the Northwestern University institutional review board, and all subjects gave informed consent.

**Table 1:** Subject demographics and clinical characteristics.

Subject	Age range	Time since stroke (years)	Lesioned hemi	Lesion location	UE FMA	Pre BBT	Post BBT	Pre AROM (°)	Post AROM (°)
S01	60–65	9	L	IC	23	0	6	−20	11
S02	60–65	8	R	IC, BG	12	1	3	0	5
S03	65–70	3	R	Par, Occ, IC	17	0	1	0	0
S04	60–65	22	R	IC, BG, Thal	11	0	1	0	17.5
S05	60–65	13	R	Occ, IC	24	0	0	0	2.5
S06	70–75	20	L	IC, BG, Thal	13	0	0	0	1.5
S07	55–60	6	L	IC, BG	24	0	3	0	5
S08	60–65	9	L	IC, Thal	22	11	13	38.5	55

*AROM, active range of motion; BBT, Box and Blocks Test; BG, basal ganglia; FMA, Fugl-Meyer Assessment; IC, internal capsule; Occ, occipital lobe; Par, parietal lobe; Thal, thalamus; UE, upper extremity*.

### Experimental Protocols

#### Intervention

Subjects participated in a 7-week intervention consisting of three 2-h visits per week. During the visit, subjects completed 20–30 trials of the following sequence of movements: (1) reaching out toward a jar, (2) driving the wrist/finger extensors to open the paretic hand, (3) grabbing the jar, (4) bringing the jar back toward themselves, and (5) releasing the jar. The weight, distance/height, and orientation of the jar relative to the subject were progressively altered to increase the challenge to each subject, as determined by the physical therapist. All subjects started the motor task with the arm supported by the table. Depending on ability, subjects were encouraged to progressively lift the paretic limb actively. During the task, a novel EMG-FES device, called ReIn-Hand, was used to assist paretic hand opening (see Figure S1 in Supplementary Material). This device recorded EMG activities from eight muscles (deltoid, biceps brachii, triceps, extensor communis digitorum, extensor carpi radialis (ECR), flexor digitorum profundus, flexor carpi radialis (FCR), and abductor pollicis). While the user performed the functional reaching and opening, the ReIn-Hand detected hand opening by extracting EMG features to trigger an Empi transcutaneous electrical neuro-stimulation device (Vista, CA, USA). The stimulation electrodes were applied to the wrist/finger extensors with the following settings: biphasic waveform, frequency = 50 Hz ± 20%, pulse width = 300 μs, amplitude = sufficient for maximal hand opening without discomfort, and duration = 3 s. The novelty of this device is that even with the increased expression of the flexion synergy at the elbow (33), wrist, and fingers (34, 35) during reaching that is prevalent in this population, the device can still detect the hand opening and drive the paretic hand open, thus allowing for a user-driven stimulation to support functional usage of the paretic hand and arm. All participants could successfully use the device to complete the described task (including opening, grasping, and releasing), although some subjects experienced difficulty in sufficiently supinating the hand when releasing the jar to keep it upright on the table. Additionally, the physical therapist stretched the hand and arm at the beginning of the experiment and between trials to effectively elicit hand openings with the EMG-FES device.

#### Pre- and Post-Intervention Tests

##### Clinical Assessments

For each subject, within 1 week prior to and following the intervention, a licensed physical therapist completed a set of clinical assessments, with the motor-related parts including UEFMA, Box and Blocks Test (BBT), and active range of motion (AROM) averaged over the II and V digit.

##### Structural Imaging of the Brain

Within 2 weeks prior to and following the intervention, subjects participated in MRI scans at Northwestern University’s Center for Translation Imaging on a 3 TS Prisma scanner with a 64-channel head coil. Structural T1-weighted scans were acquired using an MP-RAGE sequence (TR = 2.3 s, TE = 2.94 ms, FOV 256 mm × 256 mm) producing an isotropic voxel resolution of 1 mm × 1 mm × 1 mm. Visual inspection of acquired images was performed immediately following the data acquisition to guarantee no artifacts and stable head position.

##### Functional Imaging Related to Hand Opening

Within 1 week prior to and following the intervention, subjects also participated in an EEG experiment. During the EEG experiment, participants sat in a Biodex chair (Biodex Medical Systems, Shirley, NY, USA), which restrained the trunk with straps crossing the chest and abdomen. The subject’s paretic arm was placed in a forearm-hand orthosis attached to the end effector of an admittance controlled robotic device (ACT^3D^) instrumented with a six degree of freedom load cell (JR3, Inc., Woodland, CA, USA). At the beginning of each trial, subjects moved their hand to a home position, with the shoulder at 85° abduction, 40° flexion, and the elbow at 90° flexion angle. The subject then received an auditory cue. Following the cue, subjects relaxed at the home position for 5–7 s and then self-initiated a maximum attempted paretic hand opening with the arm resting on a haptic table. Subjects were instructed to avoid eye movements by focusing on a point and avoid movements of other body parts during the performance of each trial, which was visually confirmed by the experimenter. Subjects performed 60–70 trials of attempted paretic hand opening, broken into blocks (one block consisted of 20–30 trials). Rest periods varied between 15 and 60 s between trials and 10 min between blocks. The typical duration of the experiment was around 5–6 h, including ~2 h of setup, ~1 h for lunch, and ~2 h of data collection.

Scalp recordings were made with a 160-channel high-density EEG system using active electrodes (Biosemi, Inc., Active II, Amsterdam, The Netherlands) mounted on a stretchable fabric cap based on a 10/20 system. Simultaneously, EMGs were recorded from the ECR, FCR, and intermediate deltoid of the paretic arm. All data were sampled at 2,048 Hz. The impedance was kept below 5 kΩ for the duration of the experiment. Additionally, the positions of EEG electrodes on the subject’s scalp were recorded with respect to a coordinate system defined by the nasion and preauricular notches using a Polaris Krios handheld scanner and reflective markers (NDI, ON, Canada). This allowed for coregistration of EEG electrodes with each subject’s anatomical MRI data. Due to post-stroke abnormal synergy, finger/wrist extensors and flexors, and often the shoulder abductors, usually co-activate together when performing maximal hand opening (34). Therefore, in order to provide a reliable indicator of movement onset, EMGs were simultaneously recorded from the ECR, FCR, and anterior deltoid (IDL) of the paretic arm.

### Data Analysis

#### Reorganization of Cortical Activity Related to Hand Opening

EEG data were aligned to the earliest EMG onset of the three muscles and segmented from −2,200 to +200 ms (with EMG onset at 0 ms) using Brain Vision Analyzer 2 software (Brain Products, Gilching, Germany). Data were then visually inspected for the presence of artifacts. Trials exhibiting artifacts (e.g., eye blinks) were eliminated from further analysis. The remaining EEG trials were baseline-corrected (from −2,180 to −2,050 ms), low-pass-filtered at 70 Hz, and ensemble-averaged. The averaged EEG signals were down-sampled to 256 Hz and imported into CURRY 6 (Compumedics Neuroscan Ltd., El Paso, TX, USA). The cortical current density strength (μA/mm^2^) in the time between 150 and 100 ms prior to EMG onset was computed using the standardized low-resolution electromagnetic brain tomography (sLORETA) method (Lp = 1) based on a subject-specific boundary element method model with the regulation parameter automatically adjusted to achieve more than 99% variance counted (36, 37). Possible sources were located on a cortical layer with 3 mm distance between each node. Although the inverse calculation was performed over the whole cortex, only the activity in bilateral sensorimotor cortices was further analyzed. Specific regions of interest (ROI) included bilateral primary sensorimotor cortices [primary motor cortex (M1) + primary sensory cortex (S1)] and secondary motor cortices [supplementary motor area (SMA) + premotor area (PM)].

To investigate the shift of cortical activity related to hand opening, we used the estimated current density strengths to calculate a laterality index [LI = (I − C)/(I + C)], where I and C are the current density strengths from the ipsilesional and contralesional sensorimotor cortices, respectively (i.e., combined primary sensorimotor and secondary motor cortices). LI reflects the relative contributions of each cerebral hemisphere to the source activity, with a value close to +1 for an ipsilesional source distribution and −1 for a contralesional source distribution.

Additionally, we quantified a cortical activity ratio $\text{CAR}=\frac{\sum_{1}^{n}S_{n}}{\sum_{1}^{m}S_{m}}$ for each of the four ROIs, where *S* represents the current density strength of one of the nodes, and *n* and *m* represent the number of nodes in the ROI and whole sensorimotor cortices, respectively. The cortical activity ratio reflects the relative strength from one ROI as normalized by the total combined strength of the four ROIs.

#### Structural Changes in GM Density

Anatomical T1 data were analyzed with FSL-voxel-based morphometry (VBM) 1.1 (https://fsl.fmrib.ox.ac.uk/fsl/fslwiki/FSLVBM; Oxford University, Oxford, United Kingdom) (38) using FSL tools (39). First, T1 images for participants who have left hemisphere lesions were flipped to ensure that the lesions of all subjects were in the right hemisphere. The T1 images were then brain-extracted using the Brain Extraction Tool and segmented into GM using FAST4. The resulted GM partial volume images were aligned to Montreal Neurological Institute (MNI) 152 standard space using the affine registration tool FLIRT and averaged to create a study-specific GM template. Subsequently, individual GM partial volume images in native space were non-linearly registered to this template using FNIRT, modulated to correct for local expansion or contraction due to the non-linear component of the spatial transformation, and then smoothed with an isotropic Gaussian kernel with a sigma of 3 mm. Finally, a voxel-wise General Linear Model was applied with Threshold-Free Cluster Enhancement (40) to detect changes in GM density following the intervention. Voxel-based threshold of changes in GM density was set at *p* < 0.001 uncorrected.

### Statistical Analysis

Statistics were performed using SPSS (IBM, V23). Clinical and neural measures were examined for normality using a Shapiro–Wilk test. A Wilcoxon signed rank test was used if assumptions of normality were not met. A paired *t*-test was performed on LI. A 2 (time) × 4 (region) repeated measures ANOVA was performed on the cortical activity ratio. We performed *post hoc* paired *t*-tests when a main ANOVA effect was found. Significance was set at *p* < 0.05. Individual data are depicted for all significant findings.

## Results

### Changes in Arm/Hand Function following EMG-FES Task-Specific Training

Table 1 shows pre and post BBT and AROM scores. Notably, most subjects initially scored a 0 on the pre-assessment BBT and showed 0° of AROM due to the severity of their motor impairments at the arm/hand. The clinical data violated the assumptions of normality based on the Shapiro–Wilk test. Therefore, a Wilcoxon signed rank test was used and reported a significant increase in BBT following the intervention (average increase of 1.9 blocks per minute, *p* = 0.03; Table 1) and AROM (average increase of 9.9°, *p* = 0.03; Table 1), indicating improvement of paretic arm/hand control, although FMA did not change.

### Cortical Reorganization Related to the Hand

**Figure 2:**
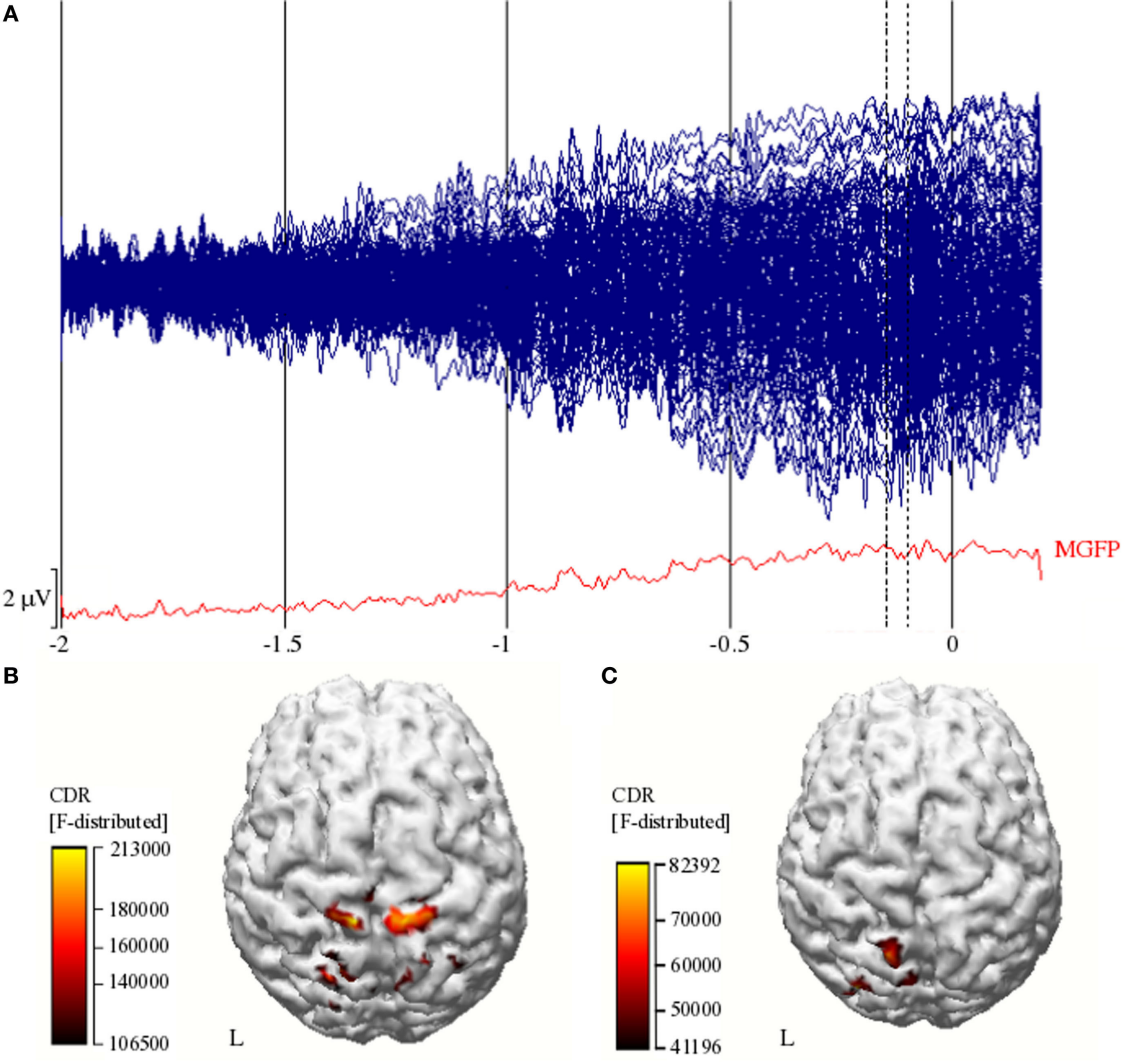
**(A)** Ensemble-averaged EEG of the 160 channels (blue butterfly plot) and Mean Global Field Power (MGFP; red line) from −2 s to +0.2 s (0 = EMG onset). Vertical dashed lines represent the start and end of the window of interest (−150 to −100 ms). A scale bar is included in the lower left; **(B)** reconstructed cortical activity between −150 and −100 ms prior to movement onset for Subject 1 during hand opening pre-intervention, and **(C)** post-intervention. Color bars indicate the current density reconstruction (CDR) statistic from sLORETA. Left hemisphere is the lesioned hemisphere.

**Figure 3:**
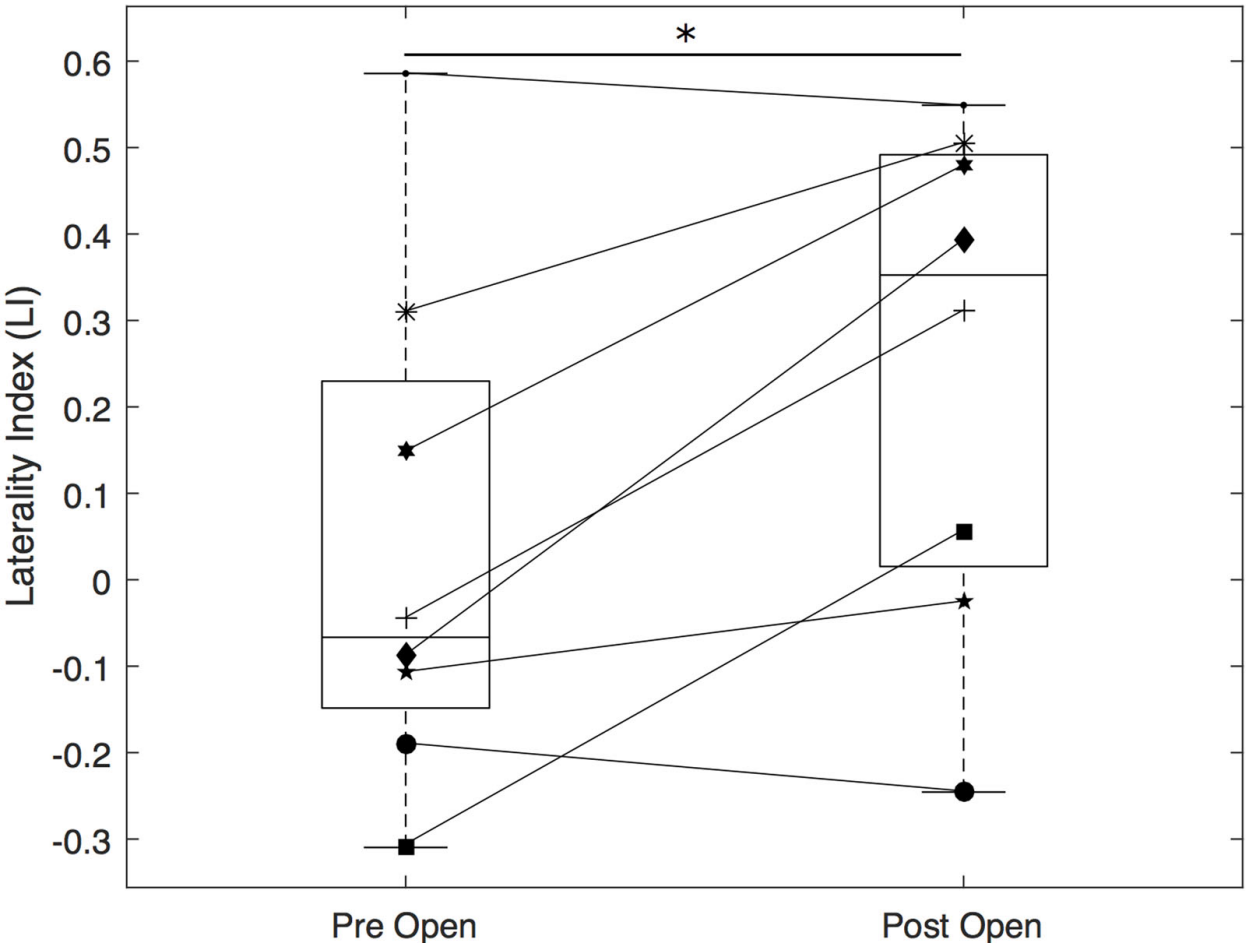
Box plots of laterality index (LI) prior to and following the intervention for paretic hand opening. Positive LI indicates predominantly ipsilesional activity. *indicates *p* < 0.05.

Figure 2A shows an example of ensemble-averaged EEG for the 160 channels for Subject 1. There is a clear baseline from roughly −2 to −1.5 s prior to EMG onset and then a slow increase in electrical potential when approaching EMG onset, consistent with the Bereitschaftspotential. The reconstructed cortical activity for Subject 1 while performing hand opening on the table is depicted in Figure 2B pre-intervention and in Figure 2C post-intervention. This subject showed bilateral activity in sensorimotor cortex prior to the intervention as seen in Figure 2B and dominant ipsilesional activity following the intervention as seen in Figure 2C. We quantified the pre- and post-intervention LI in each of the participants (see results in Figure 3). A paired *t*-test found a significant increase in LI following the intervention [*t*(7) = 3.09, *p* = 0.02], signifying a post-intervention shift toward the ipsilesional hemisphere.

**Figure 4:**
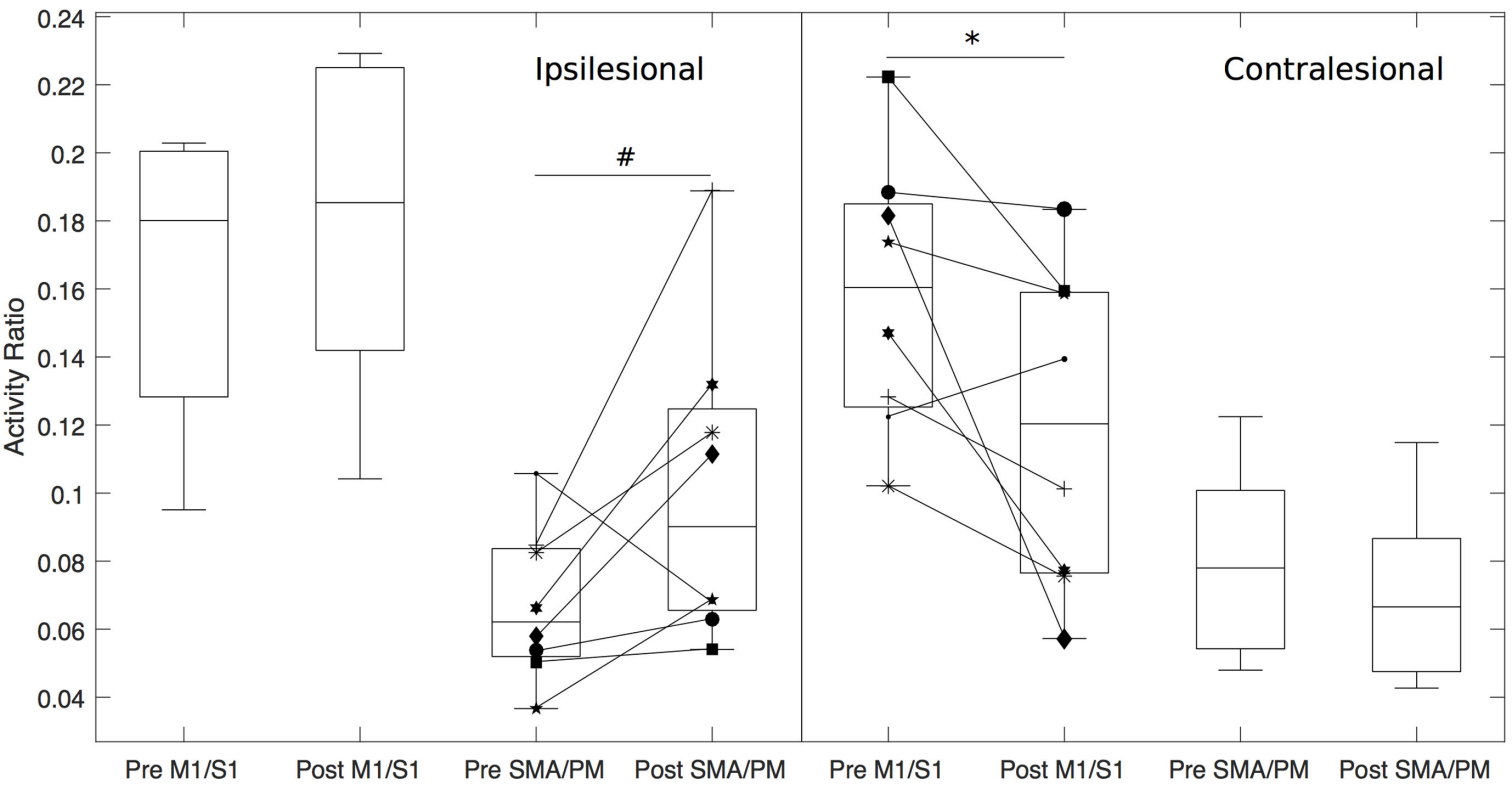
Box plots depicting cortical activity ratio prior to and following the intervention for hand opening on the table. Regions of interests include M1/S1 and supplementary motor area/premotor area (SMA/PM) for both ipsilesional (left side of figure) and contralesional (right side of figure) hemispheres. *indicates *p* < 0.05, ^#^indicates *p* = 0.06.

To further investigate regions responsible for the post-intervention LI changes, we quantified the pre- and post-intervention cortical activity ratios for primary sensorimotor (M1/S1) and secondary motor (SMA/PM) cortices (see results in Figure 4). A 2 (time) × 4 (region) repeated measures ANOVA found a significant time × region interaction [*F*(1,7) = 3.47, *p* = 0.03]. *Post hoc* paired *t*-tests found that following the intervention, there was a decrease in the cortical activation ratio in contralesional M1/S1 (*p* = 0.04) and a trending increase in ipsilesional SMA/PM (*p* = 0.06) related to paretic hand opening.

### GM Density

**Figure 5:**
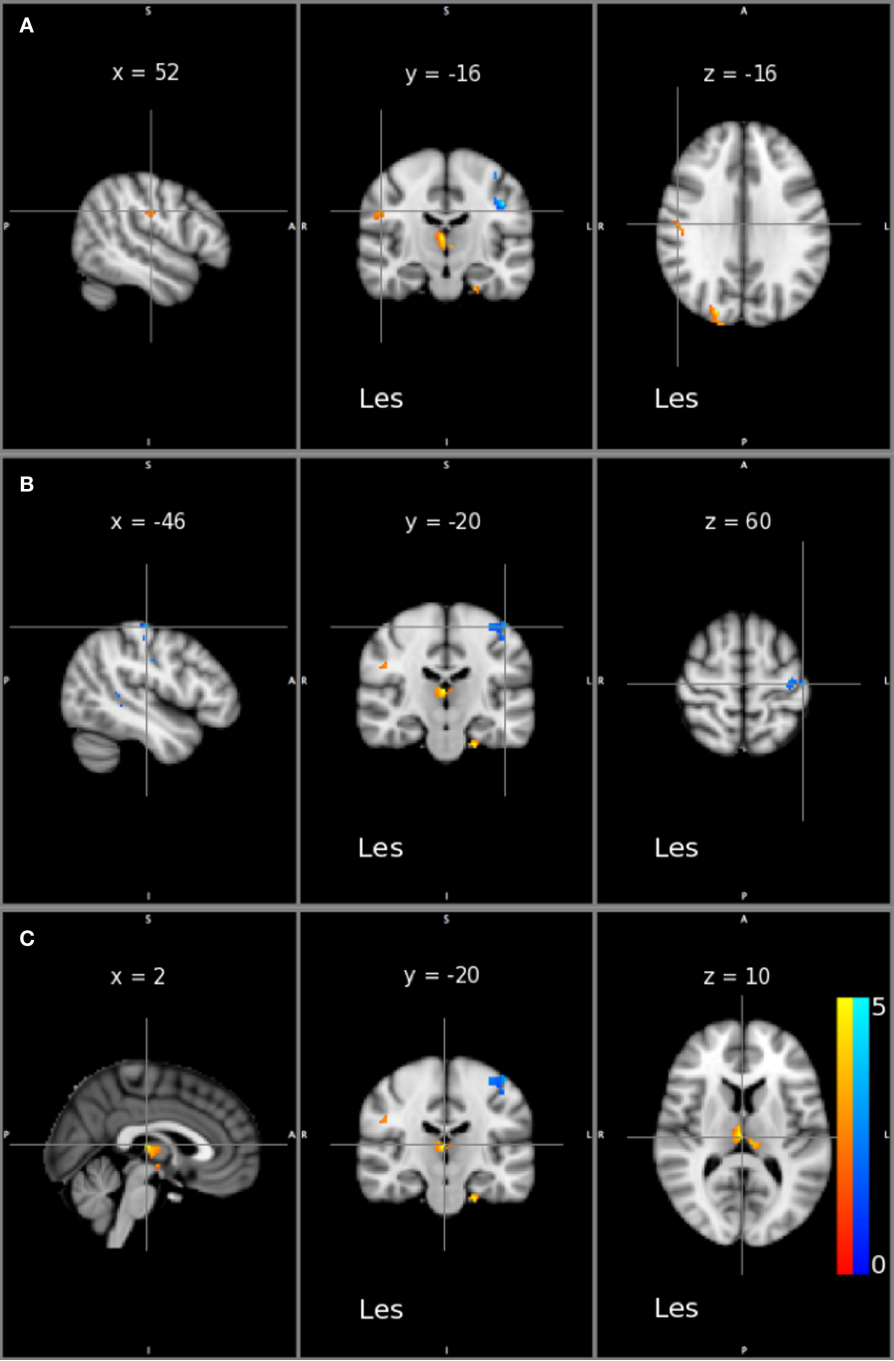
Statistical maps of gray matter (GM) density changes across all patients. Significant increases (red/yellow) and decreases (Blue) in GM density are depicted on sagittal, coronal, and axial sections (left to right) on Montreal Neurological Institute T1 slices. Sections show the maximum effect on **(A)** ipsilesioned M1/S1, **(B)** contralesional M1/S1, and **(C)** ipsilesional thalamus. Les indicates the side of the lesioned hemisphere. Color maps indicate the t values at every voxel. A statistical threshold was set at *p* < 0.001 uncorrected.

Following the intervention, subjects displayed significantly greater GM density in M1 and S1 in the lesioned hemisphere (*x* = 52, *y* = −16, *z* = 30, *t*-value = 2.55, *p* < 0.001) and a decrease in GM density in M1 and S1 in the non-lesioned hemisphere (*x* = −46, *y* = −20, *z* = 60, *t*-value = 2.41, *p* < 0.001; *x* = −44, *y* = −18, *z* = 36, *t*-value = 2.79, *p* < 0.001) as depicted in Figures 5A,B. Additionally, subjects displayed greater GM density in the thalamus in the lesioned hemisphere (*x* = 2, *y* = −20, *z* = 10, *t*-value = 3.13, *p* < 0.001) as shown in Figure 5C. A complete list of significant regions is provided in Table S1 in Supplementary Material.

## Discussion

The present study investigated neural changes in individuals with moderate to severe stroke following an EMG-FES-assisted task-specific arm/hand intervention. Specifically, we found a shift of sensorimotor cortical activity related to hand opening from contralesional to ipsilesional cortex, along with structural changes in the form of increased ipsilesional M1/S1 and decreased contralesional M1/S1 GM density. Although similar device-assisted hand/arm training in this population has been investigated before to examine behavioral improvements (7, 41, 42), this study provides evidence for corresponding neural changes even in this more severe chronic population.

### Shift toward Ipsilesional Hemisphere

As expected, before the intervention, subjects showed cortical activity predominantly from the contralesional hemisphere related to open the paretic hand, as reflected by the overall negative LI. This contralesional activity may suggest an increased reliance on low-resolution contralesional corticobulbar pathways such as the corticoreticulospinal tract (31, 43) for general paretic arm function. In fact, more severely impaired subjects actually tend to involuntarily close the hand and activate shoulder muscles when asked to open (35), which may reflect this increased reliance on ipsilateral corticobulbar pathways that innervate primarily flexor hand and proximal muscles compared to extensors (44). These pathways lack sufficient innervation to extensor muscles of the hand to produce appropriate hand opening (45) and are often associated with greater motor impairment (29, 31).

Effective hand/arm interventions in mildly impaired post-stroke individuals have reported a post-intervention shift toward ipsilesional sensorimotor areas (46, 47). This shift is thought to be a beneficial since it may indicate increased use of ipsilesional CSTs, which maintain the primary innervations to the extensor muscles of the hand (32). Intervention-induced shifts toward the ipsilesional hemisphere have rarely been investigated in more severely impaired post-stroke individuals, especially not for arm/hand training partially due to the lack of inclusion of these subjects in arm/hand interventions. In this study, we found that a ReIn-Hand-assisted arm/hand intervention induced a positive change in LI. Our results suggest that even moderate to severe chronic stroke subjects maintain the ability to show similar cortical reorganization back toward the ipsilesional hemisphere following task-specific training as seen in more mild subjects. This ipsilesional shift may suggest decreased recruitment of contralesional corticobulbar pathways and increased reliance on ipsilesional CSTs during paretic hand opening, which may allow for greater functionality at the hand as seen by the increase in BBT and AROM. Additionally, it could reflect increased ability to actually drive hand opening when instructed rather than involuntary closing and activating proximal muscles (35). It is worth noting that only six out eight participants exhibited this intervention-induced shift despite all showing improvements on either BBT or AROM, possibly reflecting compensatory behavioral strategies following the intervention rather than recovery in these two participants.

### Changes in Cortical Activity Driving LI Shift

We calculated the cortical activity ratio in each sensorimotor region to further elucidate which regions were contributing to the LI shift. Following the intervention, subjects showed decreased activity in contralesional primary sensorimotor cortex (M1/S1) and a trending increase in ipsilesional secondary motor cortex (SMA/PM).

Increased contralesional primary sensorimotor cortex activity is associated with greater impairment following stroke (48, 49) and greater damage to CST (50, 51). Therefore, this decreased activity could reflect either decreased recruitment of contralesional descending motor pathways or changes in interhemispheric balance between primary sensorimotor cortices (52) and thus allow for increased functional usage of the affected hand.

Stroke patients tend to activate secondary motor areas more following greater CST damage (51) and show positive correlations between ipsilesional secondary motor area activation and recovery (53, 54). The increased recruitment of ipsilesional SMA/PM may be due to increased recruitment of direct projections to the spinal cord (55), although these connections are not as efficacious as connections from M1 to the spinal cord (56). Alternatively, plasticity within intrinsic cortico-cortico neuronal connections in M1 (57) may allow increased communication between SMA/PM and M1 following injury. Thus, ipsilesional secondary motor areas may serve as a potential avenue for functionally relevant cortical reorganization *via* either descending or intrinsic connections in addition to removal of contralesional cortical activity.

### Increased GM Density in Ipsilesional Sensorimotor Cortex

Previous work demonstrated significant decreases in GM volume in ipsilesional precentral gyrus following a subcortical stroke, which was associated with greater impairment (58). However, following task-specific training, mild chronic stroke subjects showed increases in GM density in ipsilesional sensorimotor cortex (25), and increases in perilesional GM density were associated with better recovery in acute stroke (59). Similarly, we found increased ipsilesional M1/S1 GM density following the intervention in our moderate to severe stroke population. Additionally, a significant positive correlation was found between changes in LI and changes in GM density in ipsilesional M1/S1 following the intervention (*R*^2^ = 0.70, *p* < 0.05; Figure S2 in Supplementary Material), showing that activity shifting to the ipsilesional hemisphere was associated with increased ipsilesional M1/S1 GM density.

Increases in GM density may suggest potential synaptogenesis, dendritic growth, or gliogenesis at the cortex (60). Thus, these changes may be due to new synapse formation and dendritic growth commonly seen in animal training models (61). Additionally, these subjects likely experienced cortical atrophy prior to the intervention due to disuse of the paretic limb, which may have been partially remedied following the intervention due to increased use of the paretic arm/hand. Despite greater damage to ipsilesional descending motor tracts, these severely impaired individuals demonstrate the ability to reorganize ipsilesional primary sensorimotor cortices.

In these more severely impaired post-stroke individuals, we also found intervention-induced decreases in contralesional M1/S1 GM density, which were not reported before in mildly impaired individuals. This decrease may be specific to more severe patients since post-stroke, increased use of the contralesional hemisphere occurs to a greater degree in severely impaired individuals compared with milder individuals (29). The decrease in GM density in contralesional M1/S1 may indicate a decrease in dendritic complexity or synapses in these areas (62). These structural changes may be a result of decreased activation in these areas due to decreased recruitment during movement or overall decreased use (63, 64). Alternatively, they may be due to decreased tonic activity in these contralesional sensorimotor areas, which is thought to be a contributor to hyperexcitability in the brainstem and subsequent increased tone in this population (65, 66).

The increases in GM density seen in the thalamus in our results may be due to the repeated use of electrical stimulation throughout the intervention. Although we focused on the motor changes in this study, it is likely that these subjects show sensory neural changes as well due to the augmented afferent feedback generated by the EMG-FES device. Therefore, it is not surprising to see changes in the thalamus due to its central role as a sensory relay station for both the cutaneous and proprioceptive sensory modalities (67).

### Limitations

The main limitation of the current study is the small sample size. Despite the relatively small n, we observed consistent patterns of functional and structural changes. These changes signify the importance of examining the potential neural mechanisms found here in a larger population of moderate to severe chronic stroke subjects. Additionally, there was no control group in the present study. However, this study was aimed at investigating whether this population maintained the ability to show neural changes following an intervention, rather than answering the question of what is the optimal intervention for this population. Another potential confounding factor from the task-specific intervention is the amount of stretching. However, stretching on its own is unlikely to drive the functional and structural changes found in this study (68), even though it may temporarily reduce the stretch reflex activation of wrist and finger flexors (69). Additionally, reduced flexion synergy and subsequent decreased involuntary shoulder abduction/adduction force generation during hand opening (34) could contribute to intervention-induced changes in LI.

One of the primary long-term goals of the current study is to substantially increase the population included in task-specific therapy. Although the current ReIn-Hand device allowed our cohort of moderate to severe chronic stroke individuals to participate in task-specific training, it does require both detectable extensor EMGs to drive the device and responsiveness to FES to create sufficient hand opening. In our experience, limiting our inclusion criteria to an FMA ≥10 satisfied these requirements in most of initially screened participants (18 out of 20). However, due to the current sample size, it is difficult to accurately specify the portion of individuals who could utilize the ReIn-Hand device. However, considering that only ~5% of nearly 800 post-stroke individuals in the Clinical Neuroscience Research Registry hosted by the Rehabilitation Institute of Chicago and Northwestern University exhibit FMA scores less than 10, it clearly substantially increases coverage compared with conventional task-specific training.

## Conclusion

The present study shows the ability of even moderate to severe chronic stroke subjects to show cortical reorganization at both the functional and structural levels following a device-assisted task-specific intervention in a manner resembling that seen in mild chronic stroke subjects. Despite the tendency to focus on acute or mild chronic stroke patients in hand function rehabilitation, the current study encourages the continued push to use devices to involve moderate to severe chronic stroke subjects in task-specific arm/hand rehabilitation.

## Ethics Statement

This study was carried out in accordance with the recommendations of Northwestern University Institutional Review Board with written informed consent from all subjects. All subjects gave written informed consent in accordance with the Declaration of Helsinki. The protocol was approved by Northwestern University Institutional Review Board.

## Author Contributions

KW helped with design of the intervention and neuroimaging pre-/posttests, acquired the EEG data, ran intervention sessions, conducted EEG and VBM data analysis, contributed to interpretation, and was primary author of the manuscript. MO acquired MRI data, aided in the VBM data analysis, and contributed to interpretation and manuscript writing. CI aided in MRI data acquisition, VBM data analysis, and contributed to interpretation and manuscript writing. CC helped with the design of the intervention, ran intervention sessions, acquired the EEG data, conducted pre/post clinical assessments, conducted clinical measure data analysis, and contributed to interpretation. JD helped with the design of the intervention and neuroimaging pre-/posttests and provided interpretation to the data as well as contributions to manuscript writing. JY was the primary designer of the intervention and neuroimaging pre-/posttests, ran the intervention sessions, aided in the acquisition of the EEG data, aided in EEG analysis, provided interpretation to the data, and contributed to manuscript writing.

## Conflict of Interest Statement

The authors declare that the research was conducted in the absence of any commercial or financial relationships that could be construed as a potential conflict of interest.
