# Supplementary material for: Neural Plasticity in Moderate to Severe Chronic Stroke Following a Device-Assisted Task-Specific Arm/Hand Intervention
Source: Front Neurol. 2017 Jun 14;8:284. doi: 10.3389/fneur.2017.00284 (PMC5469871; doi:10.3389/fneur.2017.00284)
Supplement: Supplementary file 1 [file data_sheet_1.docx]

**Supplementary Material**

**Neural Plasticity in Moderate to Severe Chronic Stroke following a Task-Specific Arm/Hand Intervention**

Kevin B. Wilkins, Meriel Owen, Carson Ingo, PhD, Carolina Carmona, PT, DPT, NCS, Julius P.A. Dewald, PhD, Jun Yao, PhD*

* Correspondence: Jun Yao [j-yao@northwestern.edu](mailto:j-yao@northwestern.edu)


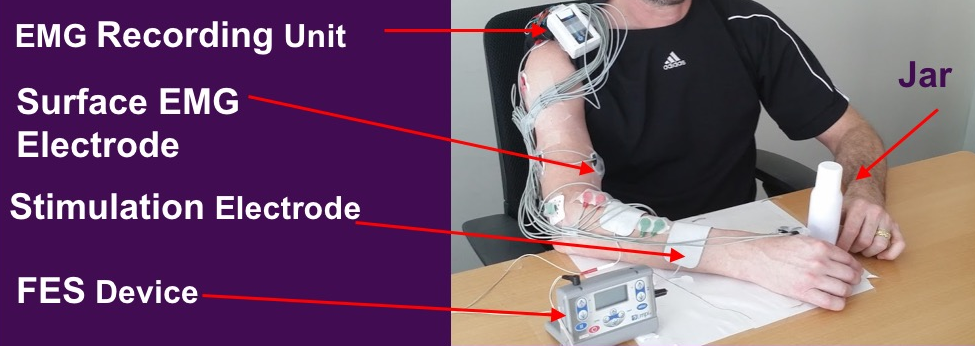
Supplementary Figure 1. Depiction of ReIn-Hand setup.

**Supplementary Table 1.** Brain regions exhibiting changes in Gray Matter Density

|  | Brain Region | Peak Voxel  MNI: x,y,z (mm) | *t* | Cluster Size (voxels) |
| --- | --- | --- | --- | --- |
| Pre > Post | L Superior Parietal Lobule | 24, -52, 44 | 3.02 | 115 |
|  | NL Postcentral Gyrus | -46, -20, 60 | 2.41 | 82 |
|  | NL Frontal Orbital Cortex | -22, 24, -24 | 3.61 | 63 |
|  | NL Precentral Gyrus | -44, -18, 36 | 2.79 | 52 |
| Post > Pre | L Thalamus | 2, -20, 10 | 3.13 | 249 |
|  | L Occipital Cortex | 22, -84, 30 | 2.58 | 112 |
|  | L Temporal Pole | 24, 12, -32 | 3.33 | 92 |
|  | NL Hippocampus | -22, -20, -32 | 3.02 | 70 |
|  | L Postcentral Gyrus | 52, -16, 30 | 2.55 | 41 |
| Note: L = Lesioned Hemisphere; NL = Non-Lesioned Hemisphere; t = t-value | | | | |

Supplementary Figure 2. Positive correlation between changes in Laterality Index (LI) and changes in Ipsilesional M1/S1 Gray Matter Density following the intervention (R^2^=0.70, p < 0.05).
